# Perirenal fat differs in patients with chronic kidney disease receiving different vitamin D-based treatments: a preliminary study

**DOI:** 10.1186/s12882-025-04041-2

**Published:** 2025-03-05

**Authors:** Ana Checa-Ros, Antonella Locascio, Owahabanun-Joshua Okojie, Pablo Abellán-Galiana, Luis D’Marco

**Affiliations:** 1 Grupo de Investigación en Enfermedades Cardiorrenales y Metabólicas, Departamento de Medicina y Cirugía, Facultad de Ciencias de la Salud, Universidad Cardenal Herrera-CEU, CEU Universities, Valencia, 46115 Spain; 2 Aston Institute of Health & Neurodeveloment (AIHN), School of Life & Health Sciences, The Aston Triangle, Aston University, Birmingham, B4 7ET UK; 3 Departamento de Ciencias Biomédicas, Facultad de Ciencias de la Salud, Universidad Cardenal Herrera-CEU, CEU Universities, Valencia, 46115 Spain

**Keywords:** Vitamin D, Analogs, Cardiovascular disease, Chronic kidney disease, Inflammation

## Abstract

**Introduction:**

Chronic kidney disease (CKD) patients show high rates of cardiovascular disease (CVD) and mortality. In the general population, obesity, hypertension, and diabetes are known as the classical CVD risk factors. However, CKD patients have other predisposing CVD factors more associated with bone and mineral metabolism disorders (BMD). BMD originates from reduced 1,25-dihydroxy vitamin D and hypocalcemia, which lead to secondary hyperparathyroidism, with increased parathyroid hormone (PTH) levels and hyperphosphatemia as the progression of renal damage. Due to their pleiotropic effects, vitamin D and its analogs, such as cholecalciferol, calcitriol, or paricalcitol, have proven effective in controlling BMD and CVD. On the other hand, visceral adiposity has been shown to increase the risk for CVD in both the general and CKD populations via complex autocrine and paracrine hormonal mechanisms. This seems to be the case with fat surrounding the epicardium. Although it has not been widely evaluated, the fat surrounding the kidneys, or the perirenal adipose tissue (PAT), could also share similarities with the epicardial in terms of its potential contribution to the CVD risk observed in these patients. We conducted a preliminary study to assess differences in PAT on a sample of patients with CKD presenting diverse CVD history and who were receiving different vitamin D-receptor activators.

**Methods/Results:**

An observational study was performed at UNIRENAL Center (Venezuela), from January to November 2015. Analytical and clinical parameters were evaluated. The PAT thickness was measured in centimeters through a B-mode ultrasound. Thus, we included 83 CKD patients treated with vitamin D or analogs (mean age 58.3 ± 16y); 57.83% were females. Nearly half of the sample was classified as CKD-G3 (*n* = 40). Prior history of CVD was present in 55.4% (*N* = 46) of participants. Must of the patients (*n* = 46;55.42%) receiving oral cholecalciferol (1000 IU/day) as part of the treatment for lower levels of vitamin D or BMD related to CKD (mainly elevated PTH), followed by those under calcitriol at 0.5 mcg/day (*n* = 27;32.53%), and around 12% (*n* = 10;12.05%) on paricalcitol (1 mcg/day). The mean treatment vintage was 20 ± 6 months for cholecalciferol, 18 ± 4 months for calcitriol, and 16 ± 2 months for paricalcitol. Those with a history of CVD (*n* = 46) showed higher levels of urea (mean 62.0vs45.2 mg/dl, *p* < 0.05), uric acid (mean 5.5vs4.3 mg/dl; *p* < 0.03), and iPTH (mean 186.2vs65.2pcg/dl; *p* < 0.05) than patients free of CVD events (*n* = 37). These findings were also in parallel with decreased renal function in the group with previous CVD history, as evidenced by a significantly lower eGFR (mean 53.55vs89.00 ml/min/1.73 m^2^,*p* < 0.001). Similarly, the mean PAT thickness was elevated in the group with a history of CVD in relation to those with no previous CVD events (0.99vs0.80 cm; SD ± 0.30;*p* ~ 0.05). The comparative analysis for the patients with prior cardiovascular events between the three treatments revealed that those on paricalcitol had lesser PAT accumulation than those treated with cholecalciferol or calcitriol (*p* < 0.05). In conclusion, our study shows that PAT thickness in CKD may be influenced by vitamin D analog-based treatment. Further research is needed to better understand the mechanistic links between PAT, BMD, and CVD in this population.

## Introduction

Patients with chronic kidney disease (CKD) show high cardiovascular complication rates [1–3], being cardiovascular disease (CVD) their main cause of morbidity and mortality [4]. Obesity, dyslipidemia, hypertension, and diabetes are known as the classical cardiovascular risk factors. However, other risk factors predisposing to CVD that appear in CKD are associated with the bone and mineral metabolism disorders (BMD) frequently present in these patients [5, 6]. BMD originates from reduced 1,25-dihydroxy vitamin D and hypocalcemia, which lead to secondary hyperparathyroidism, with increased parathyroid hormone (PTH) levels [7] and hyperphosphatemia as the kidney damage progresses [8].

Diverse therapeutic strategies are used to address BMD in CKD. Due to their pleiotropic effects, vitamin D and its analogs, such as cholecalciferol, calcitriol, or paricalcitol, have proven effective in controlling BMD secondary to CKD and cardiovascular pathologies [9, 10]. They bind to the vitamin D receptor in the parathyroid gland, thereby reducing PTH synthesis, increasing calcium absorption and the release of phosphorus from the bone [11]. As a result, they may induce hypercalcemia and hyperphosphatemia, with the consequent risk of vascular calcifications. This adverse effect is often reported with the use of calcitriol, but rarely with paricalcitol [12, 13]. On the contrary, calcimimetic agents, such as cinacalcet and etelcalcetide, which increase the sensitivity of the calcium-sensing receptor to extracellular calcium and decrease PTH secretion [14], may cause hypocalcemia [15, 16], leading to an increased risk of arrhythmia and heart failure [17].

On the other hand, visceral adiposity has been shown to increase the risk for CVD in both the general population and patients with CKD via complex autocrine and paracrine hormonal mechanisms [18]. This seems to be the case with the adipose tissue surrounding the epicardium, also known as the epicardial adipose tissue (EAT) [19]. Although it has not been widely evaluated, the adipose tissue surrounding the kidneys, or the perirenal adipose tissue (PAT), could also share similarities with the EAT in terms of its potential contribution to cardiovascular risk [20, 21].

**Fig. 1:**
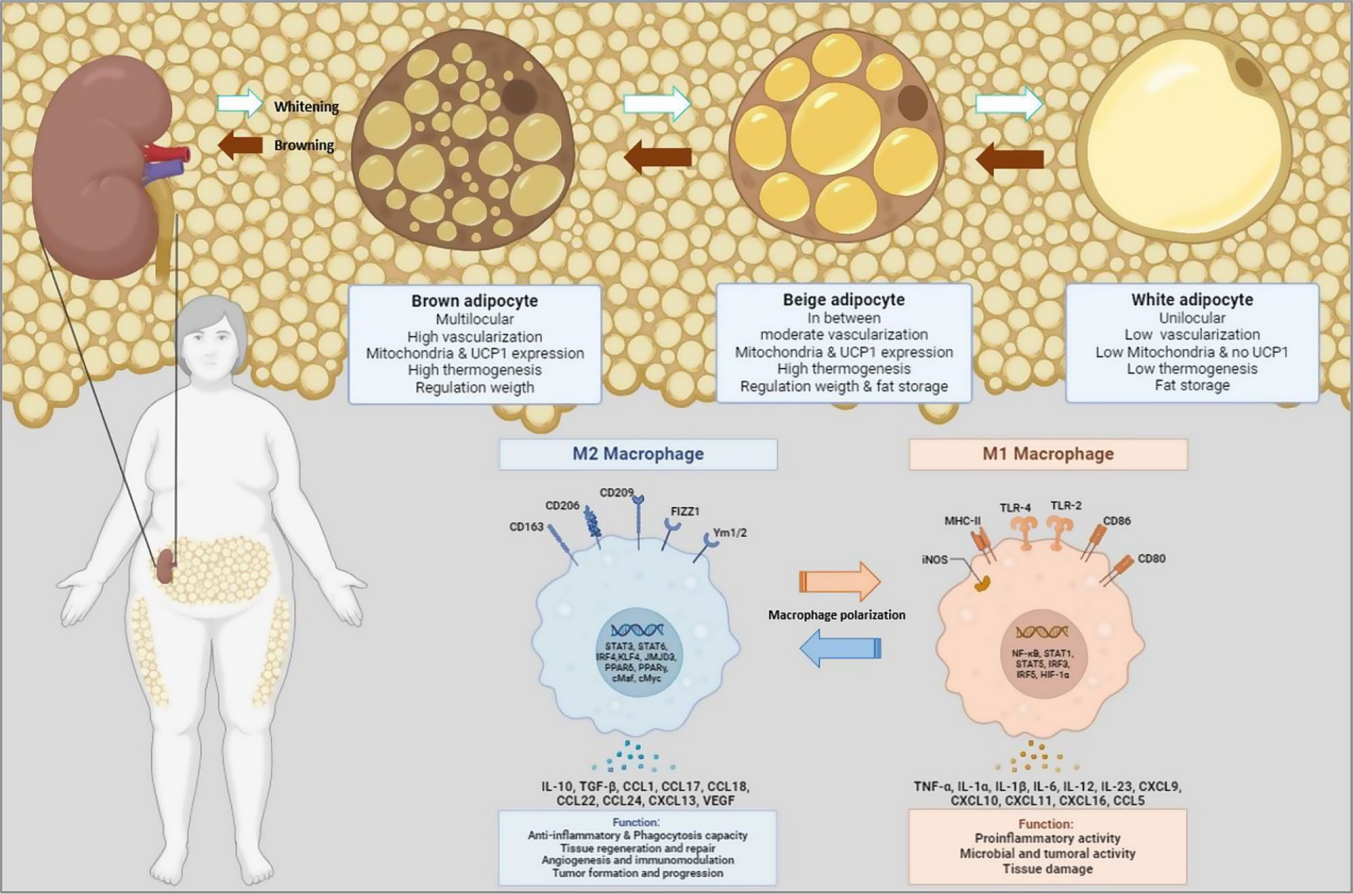
Morphological and biochemical changes of adipocytes in response to inflammatory/anti-inflammatory stimuli. The process of ‘whitening’ consists of the gradual conversion of brown adipocytes, which contain many mitochondria expressing uncoupled protein-1 (UCP-1) and are responsible for thermoregulation, into white adipocytes, whose main function is fat storage. Beige adipocytes represent an intermediate state between brown and white adipocytes. The ‘whitening’ process generally occurs in response to a pro-inflammatory environment, in which the predominant adipokine is leptin, which triggers the polarization of macrophages to an M1 phenotype. The opposite process or ‘browning’, in which the white adipocyte becomes brown, is also possible in response to anti-inflammatory stimuli: the predominant adipokine at this point is adiponectin, which prompts the macrophage polarization to an M2 phenotype

PAT, which comprises both white and brown adipose tissue, is anatomically located in the retroperitoneal space around the kidneys. It is richly vascularized by branches of the abdominal aorta, including the inferior adrenal, dorsal, and gonadal arteries [22, 23]. As a perivisceral fat, PAT is metabolically active. The specific cells from this tissue, or adipocytes, secrete several adipokines, such as adiponectin and leptin, which are cytokines involved in energy metabolism that also influence vascular function, inflammation and macrophage polarization to an anti-inflammatory or pro-inflammatory phenotype [24, 25]. Therefore, the proximity to major vascular structures and in a dysfunctional environment suggests that PAT may contribute to cardiovascular disease through various systemic and/or local mechanisms, including hormone secretion, inflammation, and lipid metabolism alterations [26, 27] (Fig. 1).

Another potential contribution of PAT to cardiovascular physiopathology is the link with the vitamin D- fibroblast growth factor 23 (FGF-23) axis. Adipocytes are capable of storing vitamin D, and vitamin D receptor activation has been shown to influence the expression of the FGF-23, a key regulator of phosphate metabolism, that has been implicated in CVD among CKD patients [28]. The dysregulation of this axis via an imbalance in the concentrations of adiponectin and leptin, which modulate FGF-23 production, may be responsible for the cardiovascular load in CKD-affected patients [29, 30].

Given the immune-regulatory properties of vitamin D and its link with cardiovascular risk factors, diverse studies focused on the morphometric and biochemical effects of vitamin D deficiency on EAT. Increased EAT thickness, in parallel with higher levels of pro-inflammatory interleukins at the EAT level, was observed in animal and clinical populations with vitamin D deficiency [31–34]. Consequently, supplementation with cholecalciferol was reported to decrease EAT thickness [35] and switch the EAT macrophage phenotype to the anti-inflammatory M2 state [36]. However, the potential association between vitamin D supplementation and PAT is scarcely explored. To date, only one study conducted on murine models revealed that those with diabetic kidney disease showed hypertrophic PAT in comparison with the controls and that vitamin D supplementation induced PAT ‘browning´, with a structural change in adipocyte mitochondria [37]. On the other hand, the differential effects induced by the diverse vitamin D analogs in either EAT or PAT are yet to be reported.

Based on the aforementioned, we hypothesized that PAT characteristics in patients with CKD could not only vary depending on their cardiovascular risk factors, but also concerning the treatment received for BMD, particularly vitamin D and analogs. Under this hypothesis, we conducted a preliminary study to assess differences in PAT thickness on a sample of patients with CKD presenting diverse CVD history and who were receiving different vitamin D-receptor activators.

## Methods

### Design

An observational study was performed at the UNIRENAL Clinical Center, Puerto Ordaz City in Venezuela, from January to November 2015. The study protocol was detailed in a prior study by our group [21], and here it is briefly summarized.

### Patient recruitment

Patients diagnosed with CKD and receiving treatment with vitamin D and analogs were the target population for this study. They needed to meet the following inclusion criteria to be invited to participate: (1) age ≥ 18 years; (2) diagnosis of CKD as per the Kidney Disease Improving Global Outcomes (KDIGO) 2012 definition (*abnormality of kidney structure or function*,* present for more than 3 months*,* with health implications*), classified as grades (G) G1 to G4 (not on dialysis); (3) treatment with vitamin D or analogs (cholecalciferol, calcitriol, paricalcitol), not in combination, initiated one year before and following the current guidelines at that moment [38] depending of 25(OH)D3 and intact (iPTH) levels (mainly iPTH levels); (4) life expectancy > 1 year. The CKD Epidemiology Collaboration (CKD-EPI) equation was used to calculate the estimated glomerular filtration rate (eGFR). Also, we used the CKD classification guidelines modified by KDIGO, which define an: eGFR (in mL/min/1.73 m^2^) > 90 as CKD G1; from 60 to 89 as CKD G2; between 30 and 59 as CKD G3; and from 15 to 29 as CKD G4.

Patients excluded from this study were those suffering from an acute inflammatory process, such as infection, active cancer, or other inflammatory states beyond the ones mentioned in inclusion, as well as subjects with acute kidney injury, polycystic kidney disease, CKD G5 (eGFR < 15 ml/min/1.73m^2^) and/or requiring renal replacement therapy.

### Clinical data collection

Electronic medical records from patients were assessed to collect the following clinical information: age; sex; eGFR and CKD grade; weight and height at recruitment onset; prior history of cardiovascular events (understood as coronary artery disease, stroke, or peripheral vascular disease); vitamin D-based treatment (cholecalciferol, calcitriol, paricalcitol), dosage and treatment duration.

Blood pressure (BP) was measured in the office at recruitment onset with a digital monitor placed on the upper arm, while the patient was sitting down with the arm on a table at the same height as the heart and after 3 min of resting (mean value of three measures).

### Analytical data collection

Blood samples to measure mineral metabolism, renal function, and inflammation markers were collected after 8 to 12 h of fasting and a 15-minute resting period, and stored at a temperature between 4 °C and 15 °C. The samples were later centrifuged in cold for 15 min and processed by absorbent photometry and turbidometry on an automatized analyzer (MINDRAY^®^ model: BS-240 China; Mindray Medical International Limited, Shenzhen, China).

### Imaging data collection

**Fig. 2:**
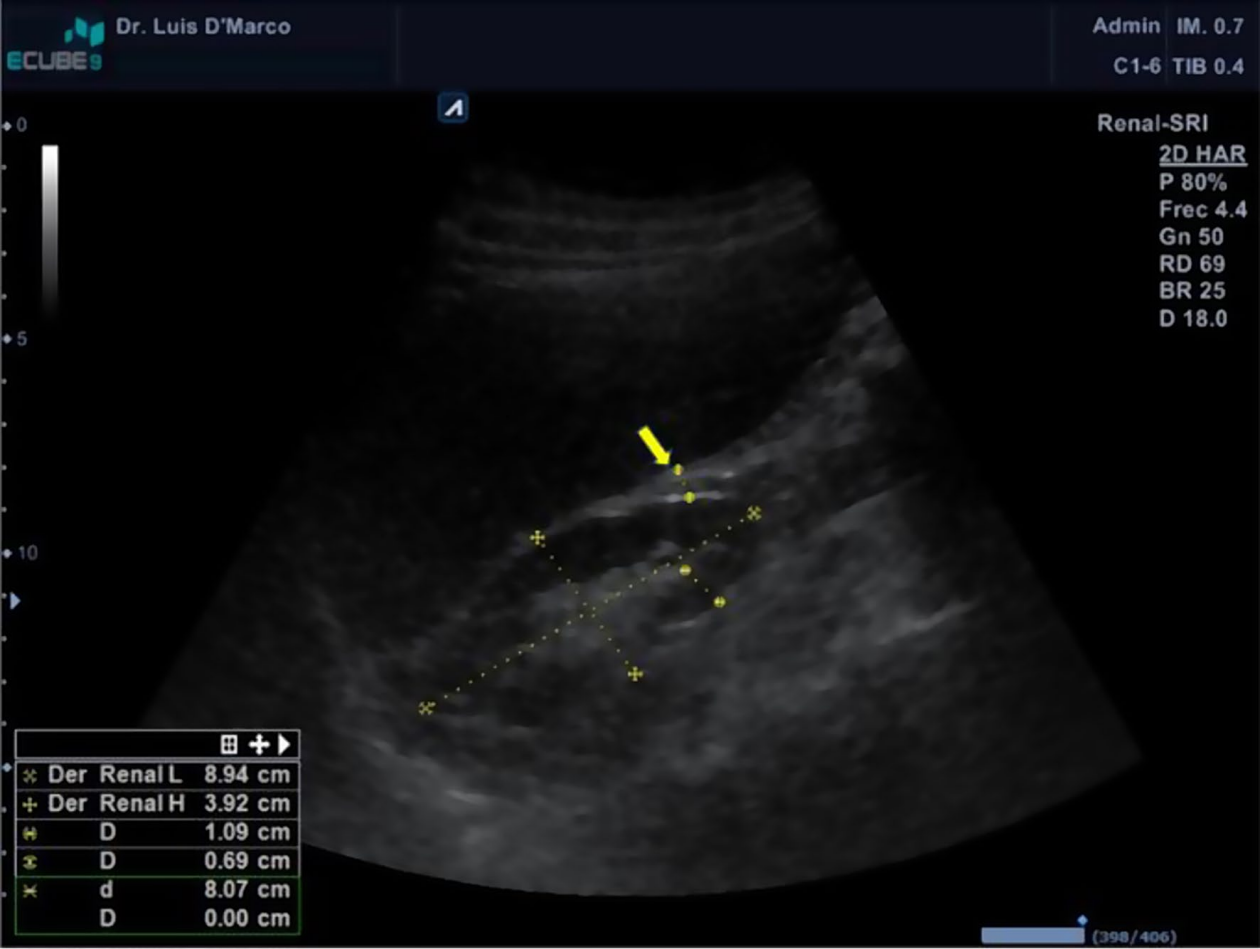
Imaging of the perirenal adipose tissue measurement by ultrasound. The arrow shows the adipose tissue thickness in the right kidney [21]

PAT thickness was measured in centimeters (cm) through a B-mode ultrasound with a 3.5-MHz convex transductor (Alpinion^®^ E-CUBE 9; Alpinion Medical Systems, Seoul, Korea). Patients underwent a bilateral renal ultrasound, and the kidneys were measured anteroposteriorly, transversally, and longitudinally. PAT was measured in the distal third between the cortex and the hepatic border and/or spleen (Fig. 2). The imaging studies were performed by one of the authors (LDM) and stored in DICOM format. Then, in the radiology department of the hospital, the imaging were processed by an expert radiologist blind to patient data.

### Ethical aspects

The research was conducted following the Declaration of Helsinki as revised in 2013. The Ethics Committee of Biomedical Research approved the study protocol. The informed consent was obtained from all patients before being included in the study.

### Statistics

The Kolmogorov-Smirnov test was used to test if the data followed a normal distribution. Descriptive data were presented as mean ± standard deviation (SD). Comparative analyses were performed through Student’s t-tests after log transformation. Comparisons of clinical, analytical, and imaging data were made between patients divided according to their prior history of cardiovascular events (history of cardiovascular disease: yes/no). PAT thickness in patients with previous CVD was later compared between the three treatment groups (cholecalciferol, calcitriol, and paricalcitol). The alpha value was set at 0.05. Calculations were performed with the SPSS 17^®^ software.

## Results

### Demographic and clinical characteristics of our sample

Our sample was composed of 83 patients with CKD under treatment with vitamin D and analogs, with a mean age of 58.3 ± 16 years, all of them of Hispanic ethnicity. Forty-eight of our patients (57.83%) were females. According to the eGFR, nearly half of our sample was classified as CKD-G3 (*n* = 40), followed by G2 (*n* = 22) and G1 (*n* = 15), with only a few patients considered as CKD-G4 (*n* = 6). Prior history of CVD was present in 46 participants (55.42%).

The majority of the patients (*n* = 46; 55.42%) were receiving oral cholecalciferol (1000 IU/day) as part of the treatment for lower levels of vitamin D or BMD related to CKD (mainly elevated iPTH), followed by those under calcitriol at 0.5 mcg/day (*n* = 27; 32.53%), and around 12% patients (*n* = 10; 12.05%) on paricalcitol (1 mcg/day). No combination of these drugs was used in any of the patients. The mean treatment duration was 20 ± 6 months for cholecalciferol, 18 ± 4 months for calcitriol, and 16 ± 2 months for paricalcitol.

### Comparisons of clinical, analytical and imaging parameters based on CVD history

Mean values of clinical, analytical, and PAT thickness are shown in Table 1, comparing patients with and without previous cardiovascular disease history. Those with a cardiovascular disease history (*n* = 46) showed significantly higher serum levels of urea (mean 62.0 vs. 45.2 mg/dl, *p* < 0.05), uric acid (mean 5.5 vs. 4.3 mg/dl; *p* < 0.03), and iPTH (mean 186.2 vs. 65.2 pcg/dl; *p* < 0.05) than the patients free of cardiovascular events (*n* = 37). These findings were also in parallel with a more decreased renal function in the group with previous CVD history, as evidenced by a significantly lower eGFR (mean 53.55 vs. 89.00 ml/min/1.73 m^2^ [CKD-EPI], *p* < 0.001). Regarding the inflammatory status, patients with prior CVD history also showed significantly higher levels of C-reactive protein (CRP) (7.1 vs. 1.2 mg/dL; *p* < 0.005). No significant differences were observed for the other analytical parameters between patients with and without CVD history.

**Fig. 3:**
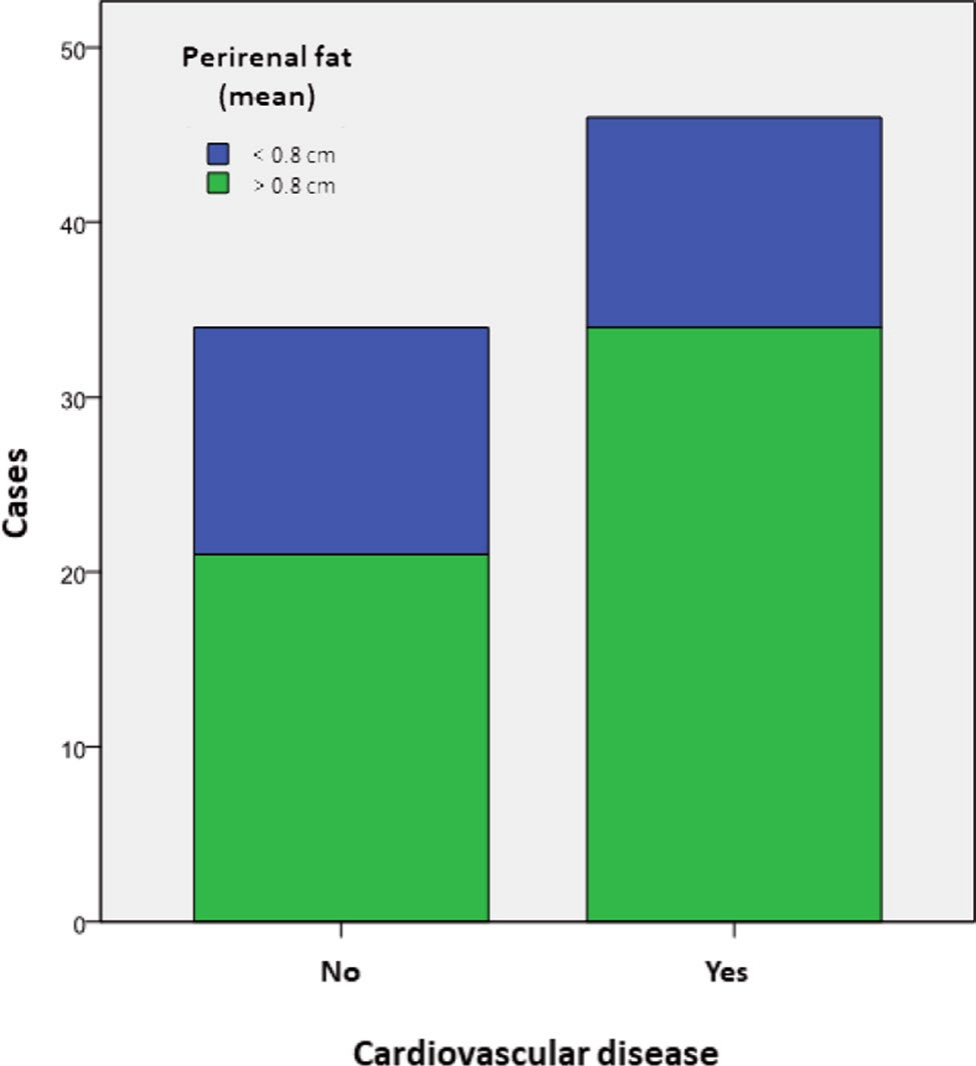
Distribution of the patients according to the history of cardiovascular disease and perirenal fat thickness

The mean PAT thickness was observed to be elevated in the group of patients with a history of CVD in relation to those with no previous cardiovascular events, although this difference did not reach statistical significance (0.99 vs. 0.80 cm; SD ± 0.30; *p* ~ 0.05) (Fig. 3).

**Fig. 4:**
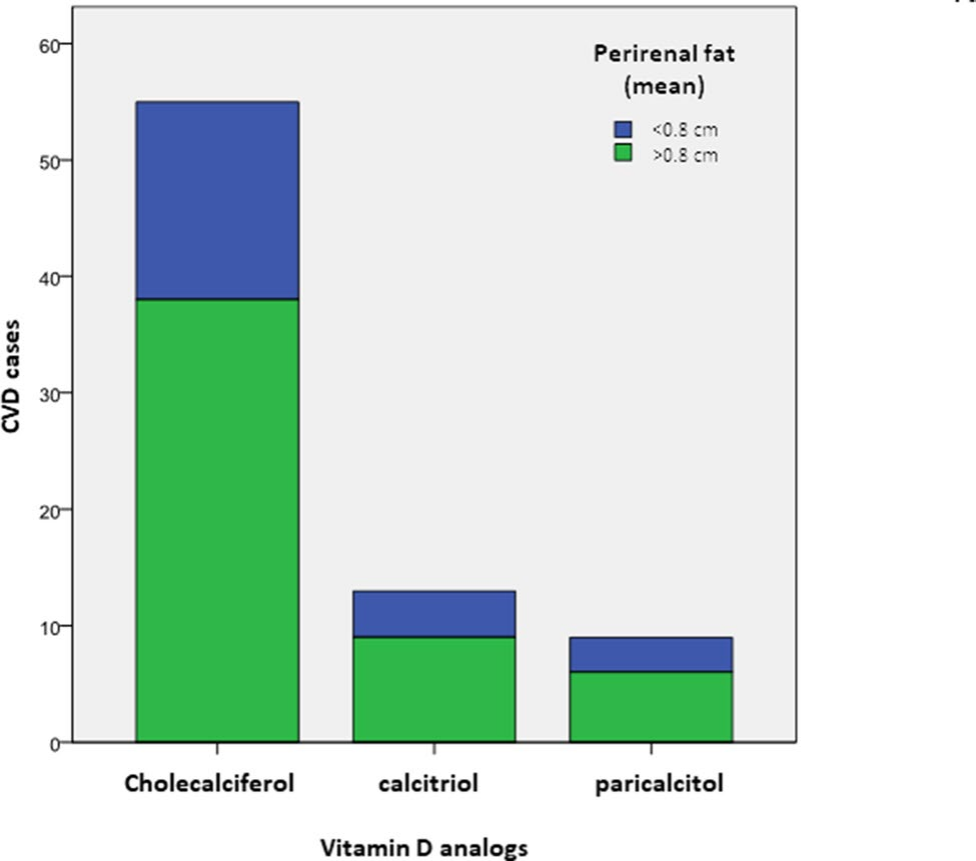
Distribution of patients according to vitamin D analogs treatments and perirenal fat thickness

When distinguishing between groups of treatment, 28 of the patients on cholecalciferol (60.87%) referred prior CVD history, whereas this occurred with 16 of the patients (59.26%) receiving calcitriol and in 2 of the patients on paricalcitol (20%). The comparative analysis for the patients with prior cardiovascular events between the three treatments revealed that those on paricalcitol had significantly lesser PAT accumulation than those treated with cholecalciferol or calcitriol (*p* < 0.05) (Fig. 4). These results were not adjusted for confounding factors such as weight, height, body mass index (BMI), and/or any other treatments.

**Table 1:** General characteristics of patients according to the history of cardiovascular disease

History of cardiovascular disease					
	Yes (*n* = 46)		No (*n* = 37)		
	Mean	SD	Mean	SD	*p*
Body mass index (kg/m^2^)	26.0	± 7.0	27.7	± 7.8	--
eGFR (mL/min/1.73 m^2^)	53.5	± 30.6	89.0	± 39.2	*< 0.001*
BP Mean (mmHg)	101.3	± 23.4	96.4	± 15.4	--
Hemoglobin (gr/dL)	12.4	± 1.3	12.5	± 1.9	--
Glucose (mg/dL)	104.5	± 19.6	102.8	± 31.3	--
Creatinine (mg/dL)	1.5	± 1.1	1.2	± 1.0	--
Urea	62.0	± 33.4	45.2	± 32.7	*< 0.05*
LDL-C (mg/dL)	106.5	± 28.9	107.9	± 31.6	--
Triglycerides (mg/dL)	131.0	± 57.4	132.3	± 73.3	--
Uric acid (mg/dL)	5.5	± 1.9	4.3	± 2.0	*< 0.03*
Albumin (gr/L)	3.7	± 0.6	4.0	± 0.5	--
Calcium (mg/dL)	9.4	± 0.6	9.4	± 0.7	--
Phosphate (mg/dL)	3.8	± 0.9	3.4	± 0.8	--
Phosphatase Alkaline (IU/L)	139.0	± 80.0	189.0	± 45.2	--
iPTH (pg/mL)	186.2	± 194.3	65.2	± 31.0	*< 0.05*
CRP (mg/dL)	7.07	± 10.5	1.29	± 1.26	*< 0.005*
PAT thickness (cm)	0.99	± 0.3	0.80	± 0.3	~ 0.05

Data are presented as mean ± standard deviation (SD). Comparisons between groups were made via the Student’s t-test after log transformation. Abbreviations: eGFR, estimated glomerular filtration rate; BP, blood pressure; LDL-C, low-density lipoprotein cholesterol; iPTH, intact parathyroid hormone; CRP, C-Reactive protein; PAT, perirenal adipose tissue

## Discussion

The results from our study suggest a potential association between PAT thickness and the presence of cardiovascular events in patients with CKD, highlighting that the role of PAT as a potential marker of cardiovascular risk needs in-depth assessment. Furthermore, the percentage of patients with prior CVD history in the paricalcitol group, which was relatively lower than with the other treatments, showed significantly decreased PAT thickness, suggesting a potential protective effect associated with this therapeutic regimen.

The finding of a thicker average PAT in patients with prior cardiovascular history raises the question about the potential role of region-specific fat storages as independent risk factors for cardiovascular damage. Although this phenomenon has been widely explored with the EAT [20, 39], the mechanisms underlying this association remain incompletely understood. As aforementioned, the adipose tissue behaves as a paracrine and endocrine organ able to influence vascular dysfunction and the inflammation status, both locally and systemically, via the secretion of different factors (adipokines) depending on the predominant adipocyte phenotype (brown/beige/white) (Fig. 1) [40].

Vitamin D deficit has been related to a greater prevalence of cancer and cardiovascular diseases [41–43]. The administration of cholecalciferol or vitamin D agonists has pleiotropic effects beyond controlling BMD, such as the regulation of immunological pathways that may be beneficial in patients with low-grade chronic inflammatory states [44, 45]. Paricalcitol is a selective vitamin D receptor agonist associated with higher efficacy, higher survival rates, and a more adequate tolerability profile than cholecalciferol or calcitriol [46–48]. Several studies have reported anti-inflammatory and antioxidant actions induced by paricalcitol that may be independent of its effects on hemodynamics and PTH suppression [49–51]. In murine models, the administration of paricalcitol was associated with reduced macrophage infiltration in the glomerular and tubular tissues after inducing renal tubular injury. On a molecular level, this occurred in parallel with decreased renal IL-6 and tumor necrosis factor-alpha (TNF-α) levels, as well as lower NADPH activity, probably via inhibition of the NLRP3 inflammasome pathway [52–54]. Although this is the first study exploring the actions of paricalcitol on PAT, the anti-inflammatory properties attributed to this drug could serve to support the hypothesized morphological and molecular actions at the PAT level.

As expected, our results show that those patients with a CVD history were more prone to higher levels of iPTH (mean 186.2 ± 194.3 vs. 65.2 ± 31.0 pg/mL; *p* 0.05) and CRP (mean 7.07 ± 10.5 vs. 1.29 ± 1.26 mg/dL; *p* < 0.005) than those free of events. Besides, those patients who have suffered prior cardiovascular events had increased uric acid serum levels (mean 5.5 ± 1.9 4.3 ± 2.0 mg/dL; *p* < 0.03). These findings are in line with the notion that altered bone and mineral metabolism are intimately related to chronic inflammatory status in renal-affected patients. Thus, careful control of these altered parameters may help to improve these conditions and avoid vascular as well as non-vascular complications.

Despite the insights provided by our study, several limitations should be acknowledged. The observational nature of our study design precludes causal inference, and the relatively small sample size limits the generalizability of our findings. Additionally, we lacked comprehensive data on more inflammatory markers and bone and mineral metabolism parameters, which may have provided further insights into the mechanisms linking PAT with cardiovascular risk in CKD patients. Regarding vitamin D serum levels, there were many missing values, and despite many patients showing higher or duplicate levels of iPTH, no vitamin D serum levels were ordered since they received direct treatment with paricalcitol depending on their iPTH serum levels.

## Conclusion

In conclusion, our study highlights that PAT thickness in CKD may be influenced by the specific vitamin D analog-based treatment used for BMD. In this regard, the use of paricalcitol could be linked to a diminishing effect on PAT thickness, which may be associated with a more favorable cardiovascular prognosis. Further research and large prospective cohort studies are needed to better understand the mechanistic links between PAT, adipokines, bone and mineral metabolism, and cardiovascular health in this population of patients. Ultimately, a deeper understanding of these pathways may open new avenues for the development of novel therapeutic strategies, such as those related to the bone-mineral, metabolic, and renal axis, to mitigate cardiovascular risk and improve outcomes in CKD patients.

## Data Availability

The datasets used and analyzed during the current study are available from the corresponding author upon reasonable request.

## References

[1] Sarnak MJ, Levey AS, Schoolwerth AC, Coresh J, Culleton B, Hamm LL, et al. Kidney disease as a risk factor for development of cardiovascular disease: A statement from the American heart association councils on kidney in cardiovascular disease, high blood pressure research, clinical cardiology, and epidemiology and prevention. Hypertension. 2003;42(5):1050–65. 14604997 10.1161/01.HYP.0000102971.85504.7c

[2] Deferrari G, Cipriani A, La Porta E. Renal dysfunction in cardiovascular diseases and its consequences. J Nephrol. 2021;34(1):137–53. 32870495 10.1007/s40620-020-00842-w PMC7881972

[3] McCullough PA, Steigerwalt S, Tolia K, Chen SC, Li S, Norris KC, et al. Cardiovascular disease in chronic kidney disease: data from the kidney early evaluation program (KEEP). Curr Diab Rep. 2011;11(1):47–55. 21076895 10.1007/s11892-010-0162-y PMC3206095

[4] Gansevoort RT, Correa-Rotter R, Hemmelgarn BR, Jafar TH, Heerspink HJL, Mann JF, et al. Chronic kidney disease and cardiovascular risk: epidemiology, mechanisms, and prevention. Lancet. 2013;382(9889):339–52. 23727170 10.1016/S0140-6736(13)60595-4

[5] Major RW, Cheng MRI, Grant RA, Shantikumar S, Xu G, Oozeerally I et al. Cardiovascular disease risk factors in chronic kidney disease: A systematic review and meta-analysis. PLoS ONE. 2018;13(3). 10.1371/journal.pone.0192895 PMC5862400 29561894

[6] Li L, Zhao J. Association of serum 25-hydroxyvitamin D with cardiovascular and all-cause mortality in patients with chronic kidney disease: NHANES 2007–2018 results. Clinics. 2024;79:100437. 38996723 10.1016/j.clinsp.2024.100437 PMC11296000

[7] Goodman WG, Quarles LD. Development and progression of secondary hyperparathyroidism in chronic kidney disease: lessons from molecular genetics. Kidney Int. 2008;74(3):276–88. 17568787 10.1038/sj.ki.5002287

[8] Cannata-Andía JB, Martín-Carro B, Martín-Vírgala J, Rodríguez-Carrio J, Bande-Fernández JJ, Alonso-Montes C, et al. Chronic kidney Disease—Mineral and bone disorders: pathogenesis and management. Calcif Tissue Int. 2021;108(4):410–22. 33190187 10.1007/s00223-020-00777-1

[9] Bover J, Ureña-Torres P, Górriz JL, Lloret MJ, da Silva I, Ruiz-García C et al. Cardiovascular calcifications in chronic kidney disease: Potential therapeutic implications. Vol. 36, Nefrologia. Elsevier Espana S.L.; 2016. pp. 597–608. 10.1016/j.nefro.2016.05.023 27595517

[10] Torres PAU, De Broe M. Calcium-sensing receptor, calcimimetics, and cardiovascular calcifications in chronic kidney disease. Kidney Int [Internet]. 2012;82(1):19–25. Available from: http://www.ncbi.nlm.nih.gov/pubmed/22437409 10.1038/ki.2012.69 22437409

[11] Portillo MR, Rodríguez-Ortiz ME. Secondary hyperparthyroidism: pathogenesis, diagnosis, preventive and therapeutic strategies. Rev Endocr Metab Disord. 2017;18(1):79–95. 28378123 10.1007/s11154-017-9421-4

[12] Shoben AB, Rudser KD, de Boer IH, Young B, Kestenbaum B. Association of oral calcitriol with improved survival in nondialyzed CKD. J Am Soc Nephrol. 2008;19(8):1613–9. 18463168 10.1681/ASN.2007111164 PMC2488261

[13] Coyne DW, Andress DL, Amdahl MJ, Ritz E, de Zeeuw D. Effects of paricalcitol on calcium and phosphate metabolism and markers of bone health in patients with diabetic nephropathy: results of the VITAL study. Nephrol Dialysis Transplantation. 2013;28(9):2260–8. 10.1093/ndt/gft227 PMC3769981 23787544

[14] Brown EM, Pollak M, Hebert SC. Sensing of extracellular Ca2 + by parathyroid and kidney cells: cloning and characterization of an extracellular Ca2+-sensing receptor. Am J Kidney Dis. 1995;25(3):506–13. 7872334 10.1016/0272-6386(95)90118-3

[15] Messa P, Maca[Combining Acute Accent]rio F, Yaqoob M, Bouman K, Braun J, von Albertini B et al. The OPTIMA Study. Clinical Journal of the American Society of Nephrology. 2008;3(1):36–45. 10.2215/CJN.03591006 PMC2390975 18178780

[16] Kilpatrick RD, Newsome BB, Zaun D, Liu J, Solid CA, Nieman K, et al. Evaluating Real-World use of Cinacalcet and biochemical response to therapy in US Hemodialysis patients. Am J Nephrol. 2013;37(4):389–98. 23548469 10.1159/000350213

[17] Goto S, Hamano T, Fujii H, Taniguchi M, Abe M, Nitta K, et al. Hypocalcemia and cardiovascular mortality in Cinacalcet users. Nephrol Dialysis Transplantation. 2024;39(4):637–47. 10.1093/ndt/gfad213 37777840

[18] Mathieu P, Poirier P, Pibarot P, Lemieux I, Després JP. Visceral obesity: the link among inflammation, hypertension, and cardiovascular disease. Hypertension [Internet]. 2009;53(4):577–84. Available from: http://www.ncbi.nlm.nih.gov/pubmed/19237685 10.1161/HYPERTENSIONAHA.108.110320 19237685

[19] Salazar J, Luzardo E, Mejías JC, Rojas J, Ferreira A, Rivas-Ríos JR, et al. Epicardial fat: physiological, pathological, and therapeutic implications. Cardiol Res Pract. 2016;2016(Cvd):1–15. 10.1155/2016/1291537 PMC4861775 27213076

[20] D’Marco L, Puchades MJ, Panizo N, Romero-Parra M, Gandía L, Giménez-Civera E et al. Cardiorenal fat: A cardiovascular risk factor with implications in chronic kidney disease. Front Med (Lausanne). 2021;(8):640814 10.3389/fmed.2021.640814 PMC8185173 34113631

[21] D’Marco L, Salazar J, Cortez M, Salazar M, Wettel M, Lima-Martínez M, et al. Perirenal fat thickness is associated with metabolic risk factors in patients with chronic kidney disease. Kidney Res Clin Pract. 2019;38(3):365–72. 31357262 10.23876/j.krcp.18.0155 PMC6727893

[22] Liu BX, Sun W, Kong XQ. Perirenal fat: A unique fat pad and potential target for cardiovascular disease. Angiology. 2019;70(7):584–93. 30301366 10.1177/0003319718799967

[23] TANUMA Y, YAMAMOTO M, ITO T. YOKOCHI C. The occurrence of brown adipose tissue in perirenal fat in Japanese. Arch Histologicum Japonicum. 1975;38(1). 10.1679/aohc1950.38.43 1200786

[24] Jespersen NZ, Feizi A, Andersen ES, Heywood S, Hattel HB, Daugaard S et al. Heterogeneity in the perirenal region of humans suggests presence of dormant brown adipose tissue that contains brown fat precursor cells. Mol Metab [Internet]. 2019;24(March):30–43. Available from: 10.1016/j.molmet.2019.03.005 10.1016/j.molmet.2019.03.005 PMC6531810 31079959

[25] Hui X, Gu P, Zhang J, Nie T, Pan Y, Wu D, et al. Adiponectin enhances Cold-Induced Browning of subcutaneous adipose tissue via promoting M2 macrophage proliferation. Cell Metab. 2015;22(2):279–90. 26166748 10.1016/j.cmet.2015.06.004

[26] Koo BK, Denenberg JO, Wright CM, Criqui MH, Allison MA. Associations of perirenal fat thickness with renal and systemic calcified atherosclerosis. Endocrinol Metabolism. 2020;35(1). 10.3803/EnM.2020.35.1.122 PMC7090296 32207272

[27] Bassols J, Martínez-Calcerrada JM, Prats-Puig A, Carreras-Badosa G, Xargay-Torrent S, Lizarraga-Mollinedo E et al. Perirenal fat is related to carotid intima-media thickness in children. Int J Obes. 2018;42(4). 10.1038/ijo.2017.236 29064476

[28] Marthi A, Donovan K, Haynes R, Wheeler DC, Baigent C, Rooney CM et al. Fibroblast growth Factor-23 and risks of cardiovascular and noncardiovascular diseases: A Meta-Analysis. J Am Soc Nephrol. 2018;29(7). 10.1681/ASN.2017121334 PMC6050929 29764921

[29] Tsuji K, Maeda T, Kawane T, Matsunuma A, Horiuchi N. Leptin stimulates fibroblast growth factor 23 expression in bone and suppresses renal 1alpha,25-dihydroxyvitamin D3 synthesis in leptin-deficient mice. J Bone Miner Res [Internet]. 2010;25(8):1711–23. Available from: http://www.ncbi.nlm.nih.gov/pubmed/20200981 10.1002/jbmr.65 20200981

[30] Spoto B, Pizzini P, Tripepi G, Mallamaci F, Zoccali C. Circulating adiponectin modifies the FGF23 response to vitamin D receptor activation: a post hoc analysis of a double-blind, randomized clinical trial. Nephrol Dial Transplant [Internet]. 2018;33(10):1764–9. Available from: http://www.ncbi.nlm.nih.gov/pubmed/29304245 10.1093/ndt/gfx344 29304245

[31] Chen X, Wu W, Wang L, Shi Y, Shen F, Gu X, et al. Association between 25-Hydroxyvitamin D and epicardial adipose tissue in Chinese Non-Obese patients with type 2 diabetes. Med Sci Monit. 2017;23:4304–11. 28877159 10.12659/MSM.904755 PMC5598744

[32] Gurses KM, Tokgozoglu L, Yalcin MU, Kocyigit D, Evranos B, Yorgun H, et al. Epicardial fat thickness is increased in vitamin D deficient premenopausal women and does not decrease after Short-term replacement. J Atheroscler Thromb. 2015;22(6):582–9. 25739691 10.5551/jat.28381

[33] Gupta GK, Agrawal T, DelCore MG, Mohiuddin SM, Agrawal DK. Vitamin D deficiency induces cardiac hypertrophy and inflammation in epicardial adipose tissue in hypercholesterolemic swine. Exp Mol Pathol. 2012;93(1):82–90. 22537546 10.1016/j.yexmp.2012.04.006 PMC3411274

[34] Dozio E, Briganti S, Vianello E, Dogliotti G, Barassi A, Malavazos AE, et al. Epicardial adipose tissue inflammation is related to vitamin D deficiency in patients affected by coronary artery disease. Nutr Metabolism Cardiovasc Dis. 2015;25(3):267–73. 10.1016/j.numecd.2014.08.012 25315671

[35] Sunbul M, Bozbay M, Mammadov C, Cincin A, Atas H, Ozsenel EB, et al. Effect of vitamin D deficiency and supplementation on myocardial deformation parameters and epicardial fat thickness in patients free of cardiovascular risk. Int J Cardiovasc Imaging. 2015;31(4):765–72. 25697721 10.1007/s10554-015-0622-1

[36] Gunasekar P, Swier VJ, Fleegel JP, Boosani CS, Radwan MM, Agrawal DK. Vitamin D and macrophage polarization in epicardial adipose tissue of atherosclerotic swine. PLoS ONE. 2018;13(10):e0199411. 30296271 10.1371/journal.pone.0199411 PMC6175496

[37] Zheng X, Huang Y, Yang M, Jin L, Zhang X, Zhang R, et al. Vitamin D is involved in the effects of the intestinal flora and its related metabolite TMAO on perirenal fat and kidneys in mice with DKD. Nutr Diabetes. 2024;14(1):42. 38858392 10.1038/s41387-024-00297-z PMC11164932

[38] Eckardt KU, Kasiske BL. KDIGO clinical practice guideline for the diagnosis, evaluation, prevention, and treatment of chronic kidney Disease-Mineral and bone disorder (CKD-MBD). Kidney Int. 2009;76:S1–2. 10.1038/ki.2009.188 19644521

[39] Karohl C, D’Marco L, Bellasi A, Raggi P. Hybrid myocardial imaging for risk stratification prior to kidney transplantation: added value of coronary calcium and epicardial adipose tissue. J Nuclear Cardiol. 2013;20(6):1013–20. 10.1007/s12350-013-9761-8 24026479

[40] A new Article. Series for adipose tissue. Nat Rev Endocrinol. 2023;19(5):249–249. 36997807 10.1038/s41574-023-00832-5

[41] Rojas-Rivera J, De La Piedra C, Ramos A, Ortiz A, Egido J. The expanding spectrum of biological actions of vitamin D. Nephrol Dial Transplant [Internet]. 2010;25(9):2850–65. Available from: http://www.ncbi.nlm.nih.gov/pubmed/20525641 10.1093/ndt/gfq313 20525641

[42] Skaaby T, Husemoen LLN, Thuesen BH, Pisinger C, Jørgensen T, Roswall N et al. Prospective population-based study of the association between serum 25-hydroxyvitamin-D levels and the incidence of specific types of cancer. Cancer Epidemiol Biomarkers Prev [Internet]. 2014;23(7):1220–9. Available from: http://www.ncbi.nlm.nih.gov/pubmed/24789846 10.1158/1055-9965.EPI-14-0007 24789846

[43] Anderson JL, Vanwoerkom RC, Horne BD. Parathyroid hormone, vitamin D, renal dysfunction, and cardiovascular disease: Dependent or independent risk factors? Am Heart J [Internet]. 2011;162(2):331–339.e2. Available from: 10.1016/j.ahj.2011.05.005 10.1016/j.ahj.2011.05.005 21835295

[44] Wang TT, Nestel FP, Bourdeau V, Nagai Y, Wang Q, Liao J, et al. Cutting edge: 1,25-Dihydroxyvitamin D3 is a direct inducer of antimicrobial peptide gene expression. J Immunol. 2004;173(5):2909–12. 15322146 10.4049/jimmunol.173.5.2909

[45] Gombart AF. The vitamin D–Antimicrobial peptide pathway and its role in protection against infection. Future Microbiol. 2009;4(9):1151–65. 19895218 10.2217/fmb.09.87 PMC2821804

[46] Ong LM, Narayanan P, Goh HK, Manocha AB, Ghazali A, Omar M, et al. Randomized controlled trial to compare the efficacy and safety of oral paricalcitol with oral calcitriol in dialysis patients with secondary hyperparathyroidism. Nephrology. 2013;18(3):194–200. 23311404 10.1111/nep.12029

[47] Jamaluddin EJ, Gafor AHA, Yean LC, Cader R, Mohd R, Kong NCT, et al. Oral paricalcitol versus oral calcitriol in continuous ambulatory peritoneal dialysis patients with secondary hyperparathyroidism. Clin Exp Nephrol. 2014;18(3):507–14. 23903802 10.1007/s10157-013-0844-2

[48] Ketteler M, Martin KJ, Wolf M, Amdahl M, Cozzolino M, Goldsmith D, et al. Paricalcitol versus Cinacalcet plus low-dose vitamin D therapy for the treatment of secondary hyperparathyroidism in patients receiving haemodialysis: results of the IMPACT SHPT study. Nephrol Dialysis Transplantation. 2012;27(8):3270–8. 10.1093/ndt/gfs018 PMC3408938 22387567

[49] Navarro-González JF, Donate-Correa J, Méndez ML, de Fuentes MM, García-Pérez J, Mora-Fernández C. Anti-inflammatory profile of paricalcitol in hemodialysis patients: a prospective, open-label, pilot study. J Clin Pharmacol [Internet]. 2013;53(4):421–6. Available from: http://www.ncbi.nlm.nih.gov/pubmed/23426718 10.1002/jcph.19 23426718

[50] Alborzi P, Patel NA, Peterson C, Bills JE, Bekele DM, Bunaye Z et al. Paricalcitol reduces albuminuria and inflammation in chronic kidney disease: a randomized double-blind pilot trial. Hypertension [Internet]. 2008;52(2):249–55. Available from: http://www.ncbi.nlm.nih.gov/pubmed/18606901 10.1161/HYPERTENSIONAHA.108.113159 18606901

[51] D’Marco L, Checa-Ros A, Gamero D, Soto C, Salazar J, Nava M, et al. Etelcalcetide and paricalcitol in chronic kidney disease: when the target is inflammation. Healthcare. 2022;11(1):72. 36611532 10.3390/healthcare11010072 PMC9818894

[52] Cirilo MA, de Ribeiro S, Lima FPB, Silva NK dos, Gomes JK, Albuquerque JA et al. JSS,. Paricalcitol prevents renal tubular injury induced by ischemia-reperfusion: Role of oxidative stress, inflammation and AT1R. Mol Cell Endocrinol. 2024;594:112349. 10.1016/j.mce.2024.112349 39233041

[53] Deluque AL, de Almeida LF, Oliveira BM, Souza CS, Maciel ALD, Francescato HDC, et al. Paricalcitol prevents MAPK pathway activation and inflammation in adriamycin-induced kidney injury in rats. J Pathol Transl Med. 2024;58(5):219–28. 39183499 10.4132/jptm.2024.07.12 PMC11424196

[54] Huang J, Zhang P, An Q, He L, Wang L. New insights into the treatment mechanisms of vitamin D on PM2.5-induced toxicity and inflammation in mouse renal tubular epithelial cells. Int Immunopharmacol. 2022;108:108747. 35429817 10.1016/j.intimp.2022.108747

